# A complete clinico-epidemiological and microbiological profile of candidemia cases in a tertiary-care hospital in Western India

**DOI:** 10.1017/ash.2021.235

**Published:** 2022-03-07

**Authors:** Ekadashi Rajni, Preeti Chaudhary, Vishnu Kumar Garg, Rajani Sharma, Manisha Malik

**Affiliations:** 1 Department of Microbiology, Mahatma Gandhi University of Medical Sciences & Technology, Jaipur, Rajasthan, India; 2 Department of Anaesthesiology, Mahatma Gandhi University of Medical Sciences & Technology, Jaipur, Rajasthan, India; 3 Department of Microbiology, Government Medical College, Bhilwara, Rajasthan, India; 4 Department of PSM (Community Medicine), Churu Medical College, Churu, Rajasthan, India

## Abstract

**Objective:**

To identify different *Candida* spp along with antifungal susceptibility pattern and risk factors associated with candidemia.

**Design, setting, and patients:**

This retrospective observational study was conducted in a tertiary-care academic hospital in Jaipur, Western India, for 3 years (July 2017–June 2020).

**Methods:**

Blood cultures were performed according to standard microbiological methods, and only 1 isolate per patient was included in the study. Isolates of *Candida* spp were identified using a VITEK-2 automated system and matrix-assisted laser desorption ionization-time of flight mass spectrometry. Antifungal susceptibility tests were performed using the broth microdilution assay according to the Clinical and Laboratory Standards Institute guidelines.

**Results:**

Of 3,443 blood cultures received from suspected sepsis cases, candidemia was identified in 95 (2.8%). In addition to *Candida tropicalis* (n = 36; 38%) and *Candida parapsilosis* (n = 17; 18%), 10 isolates of *Candida auris* comprised the fourth most common cause of candidemia. Presence of central venous catheter and diabetes were statistically significant risk factors for development of candidemia by NAC. Resistance to fluconazole was 36%, resistance to voriconazole was 20%, resistance to 5-flucytosine was 4%, and resistance to amphotericin-B was 7%. *C. auris* isolates were more resistant than other NAC spp. We detected no resistance among the echinocandins.

**Conclusions:**

The emergence of highly resistant isolates like *C. auris* emphasizes the need for constant monitoring of candidemia cases for species identification and routine antifungal susceptibility so that appropriate measures can be taken to reduce the related morbidity and mortality.

The term candidemia refers to the presence of *Candida* spp in the blood. It is the most common form of invasive candidiasis among hospitalized patients. It is associated with high crude and attributable mortality rates along with increased duration of hospitalization and cost of care. Although earlier studies have ranked *Candida* species as the seventh most common causative organism of healthcare-associated bloodstream infection (BSI),^1^ more recent studies place them third or fourth.^2,3^

The evolution of complex medical and surgical procedures undertaken to prolong the survival of critically ill patients, coupled with usage of antibiotics and antifungals for elongated periods, has led to an increase in the incidence of candidemia. Simultaneously, a massive shift in landscape of candidemia is being reported on a global level. Although *C. albicans* is still a common causative species, increasing rates of candidemia caused by *C. tropicalis*, *C. parapsilosis*, *C. glabrata*, and *C. krusei* have been reported worldwide.^4–7^ The emerging fungal pathogen *C. auris* is causing outbreaks of invasive disease in healthcare facilities around the world.^8,9^ Furthermore, growing resistance to commonly used first- and second-line antifungals (ie, fluconazole and echinocandins) represents a major challenge for empirical and prophylactic strategies.

The epidemiology of candidemia varies remarkably according to geographic regions. *Candida albicans* is the predominant species causing candidemia in Malaysia, Singapore, Thailand, Australia, and Japan, but *C. tropicalis* is predominant on the Indian subcontinent and neighboring countries.^10^ Continuous surveillance is thus mandatory for monitoring trends in local incidence, species distribution, and antifungal susceptibility profiles.

Data pertaining to contemporary epidemiology of candidemia from Western India are sparse. Our comprehensive study addresses issues like the prevalence of candidemia, its etiology, and the antifungal susceptibility profiles from Jaipur, Rajasthan. In addition, this study also provides insight into associated risk factors and susceptibility profiles between *C. albicans* and non-*albicans Candida* (NAC) cases. To the best of our knowledge, no previous study from Rajasthan has addressed these critical issues. The information gained from this study should help both clinicians and microbiologists in developing an empirical antifungal therapy module and aggressively handling candidemia cases.

## Methods

This retrospective, single-center observational study was conducted in a 1,500-bed tertiary-care private sector hospital in Jaipur, Western India, for 3 years (July 2017–June 2020). Candidemia is defined as isolation of any *Candida* spp from 1 or more blood cultures of a patient in the presence of clinical features of sepsis. All blood culture results of suspected sepsis cases during the study period at our center were screened and all patients diagnosed with candidemia were included in the study. Only 1 isolate per patient was included.

Blood culture was performed using an automated method (BACTEC 9050, Becton Dickinson, Franklin Lakes, NJ). Isolates of *Candida* spp were identified using a VITEK-2 automated system (bioMèrieux, Marcy-l’Étoile, France) with VITEK 2 (YST) cards. *Candida* isolates that could not be identified conclusively by VITEK-2 were subjected to matrix-assisted laser desorption ionization-time of flight mass spectrometry (MALDI-TOF MS; Bruker Biotyper OC version 3.1, Daltonics, Bremen, Germany) using an ethanol formic acid extraction protocol. Antifungal susceptibility tests were performed using the broth microdilution assay according to the Clinical and Laboratory Standards Institute guidelines.^11^

Electronic patient records were reviewed from hospital information management system. Patient data regarding duration of hospital stay, demographic details, baseline characteristics, comorbid illnesses, laboratory findings, clinical outcome or any medical procedures performed were obtained and tabulated in an Excel worksheet (Microsoft, Redmond, WA).

### Statistical analysis

The data were cleaned and coded. Prevalence was measured as cases per 100 samples along with 95% confidence limits. Demographic and clinical characteristics of patient were expressed as numbers and percentages for categorical variables. The association of these variables with *C. albicans* was analyzed using the χ^2^ test. Comparison of antifungal susceptibility between NAC and *C. albicans* was inferred using the χ^2^ test results. All analyses were conducted using SPSS version 20 software (IBM, Armonk, NY). A *P* value < .05 was considered statistically significant.

Approval for this study was obtained from our institutional ethical committee (no. MGMCH/IEC/JPR/2020/182).

## Results

**Fig. 1.**
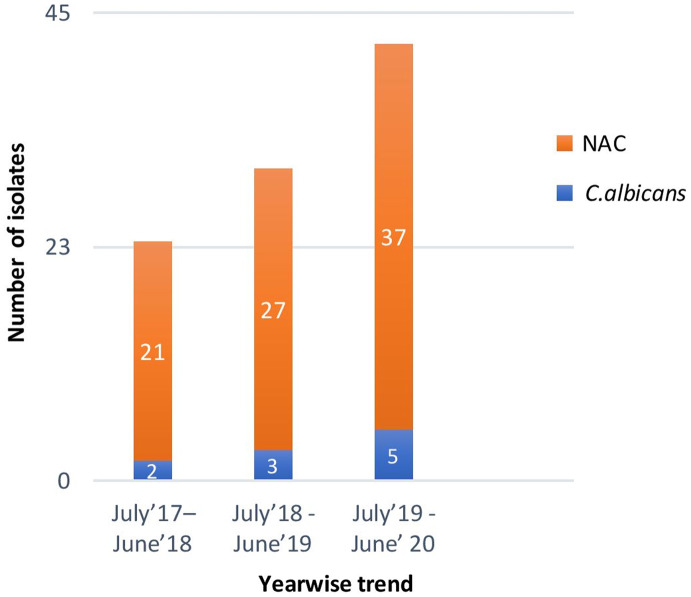
Year-wise rising trend of candidemia over the 3-year study period.

In total, 3,443 blood samples from suspected sepsis cases were received at our center during the 3-year study period, and *Candida* spp were isolated in 95 (2.8%) samples. An upward trend in *Candida* spp isolation was noted over the 3-year study period. The prevalence of candidemia in sepsis cases increased from 23 cases (0.7%) in July 2017–June 2018 to 30 cases (0.9%) in July 2018–June 2019 and to 42 cases (1.2%) in July 2019–June 2020 (Fig. 1). The prevalence of candidemia was significantly higher in the ICU compared to other wards: 79% vs 21% (*P* < .001).

Table 1 shows the distribution of various *Candida* spp isolated from blood. Of the 95 *Candida* strains isolated, the prevalence of NAC (n = 85, 89%; 95% confidence interval [CI], 81.7%–94.2%) was higher than that of *C. albicans* (n = 10, 11%). Among the NAC, *C. tropicalis* (n = 36, 38%) was the most common isolate, followed by *C. parapsilosis* (n = 17, 18%), *C. famata* (n = 11, 12%), *C. auris* (n = 10, 11%) and *C. glabrata* (n = 5, 5%).

**Table 1.** Distribution of Various *Candida* spp Isolated From Blood

Species	No. (%)
*Candida tropicalis*	36 (38)
*C. parapsilosis*	17 (18)
*C. famata*	11 (12)
*C. auris*	10 (11)
*C. albicans*	10 (11)
*C. glabrata*	5 (5)
*C. rugosa*	1 (1)
*C. lusitanae*	1 (1)
*C. lipolytica*	1 (1)
*C. utilis*	1 (1)
*C. pelliculosa*	1 (1)
*C. guillermondii*	1 (1)
Total	95 (100)

### Clinico-epidemiological features

The clinical and demographic features of candidemia cases are summarized in Table 2 which shows a male preponderance with a male-to-female ratio of 2:1. Most cases were observed in the group aged 19–60 years. No statistically significant difference was noted in sex or age-group distribution between cases caused by *C. albicans* and NAC.

**Table 2.** Clinical Epidemiological Characteristics and Risk Factor Distribution Among Various *Candida* spp Causing Candidemia

Parameter	*Candida albicans*, No. (%)	Non-*albicans Candida*, No. (%)	Total, No. (%)	*P* Value
**Age, y**				
0–18	…	7 (7.36)	7 (7.36)	.92
19–60	4 (4.21)	56 (58.94)	60 (63.15)	.92
>60	6 (6.31)	22 (23.15)	28 (29.47)	.84
**Sex**				
Female	3 (3.15)	29 (30.52)	32 (33.68)	.98
Male	7 (7.36)	56 (58.94)	63 (66.31)	.98
**Patient location**				
ICU	7 (7.36)	68 (71.57)	75 (78.94)	.97
Wards	3 (3.15)	17 (17.89)	20 (21.05)	.94
**Risk factor**				
CVC	8 (8.42)	39 (41.05)	47 (49.47)	.04
TPN	2 (2.1)	11 (11.57)	13 (13.68)	.53
Diabetes	8 (8.42)	36 (37.89)	44 (46.31)	.04
Hypertension	4 (4.21)	31 (32.63)	35 (36.84)	.82
Cardiovascular diseases	3 (3.15)	11 (11.57)	14 (14.73)	.16
Chronic kidney diseases	2 (2.1)	18 (18.94)	20 (21)	.93
HIV	…	6 (6.31)	6 (6.31)	.39
Solid organ transplantation	…	1 (1.05)	1 (1.05)	.72
Lung diseases	2 (2.1)	19 (20)	21 (22.1)	.09
Malignancy	3 (3.15)	9 (9.47)	12 (12.63)	.08
History of blood transfusion	1 (1.05)	28 (29.47)	29 (30.52)	.13
Urinary catheter	2 (2.1)	41 (43.15)	43 (45.26)	.09
Invasive ventilation	5 (5.26)	24 (25.26)	29 (30.52)	.16
Surgery within 3 mo	4 (4.21)	37 (38.94)	41 (43.15)	.83
Broad-spectrum antibiotics	7 (7.36)	58 (61.05)	65 (68.42)	.91
Steroid therapy	4 (4.21)	28 (29.47)	32 (33.68)	.30
**Patient outcome**				
Overall mortality	3 (3.15)	21 (22.1)	24 (25.26)	.71

Note. ICU, intensive care unit; CVC, central venous catheter; TPN, total parenteral nutrition; HIV, human immunodeficiency virus.

The most common risk factor associated with candidemia was broad-spectrum antibiotic usage (68%), followed by presence of central venous catheter (49%), urinary catheterization (45%), and corticosteroid therapy (34%). Comorbid diabetes was present in 46% of patients, and comorbid hypertension was present in 37%. The presence of a central venous catheter and diabetes were statistically higher in cases of candidemia caused by NAC in our study (*P* = .04) (Table 2).

Of the 95 candidemia cases, 24 (25%) succumbed to infection, whereas 42 (44%) recovered completely. Clinical outcomes could not be ascertained for 29 patients (31%) because they left against medical advice.

### Antifungal susceptibility

**Fig. 2.**
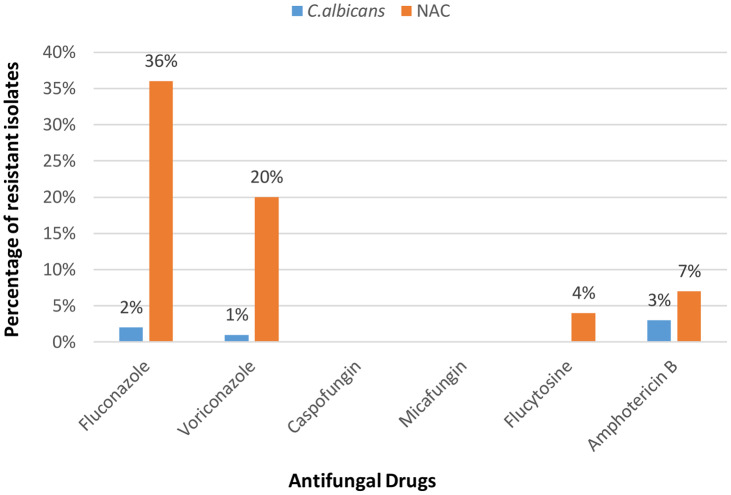
Resistance pattern of *Candida* spp (%) to different antifungal drugs.

Figure 2 shows the antifungal susceptibility pattern to various drugs tested. *C. albicans* were 100% sensitive to caspofungin (CAS), micafungin (MIC), and 5-flucytosine (5-FC). Resistance to fluconazole (FLC) was observed in 2%, resistance to voriconazole (VRC) was observed in 1%, and resistance to amphotericin B was observed and 3%. Among the NAC isolates, 100% sensitivity was noted for the echinocandins (caspofungin and micafungin). Resistance to amphotericin B was statistically higher among NAC isolates (*P* = .03) (Table 3).

**Table 3.** Comparison of Antifungal Susceptibility Between Non-*albicans Candida* and *Candida albicans*

	No. of Isolates					
Species	Fluconazole	Voriconazole	Caspofungin	Micafungin	Amphotericin B	5-Flucytosine
*Candida albicans*	2	1	Nil	Nil	3	Nil
NAC	34	19	Nil	Nil	7	4
*P* value	.31	.68	…	…	.03	1.00

Note. NAC, non-*albicans Candida.*

**Fig. 3.**
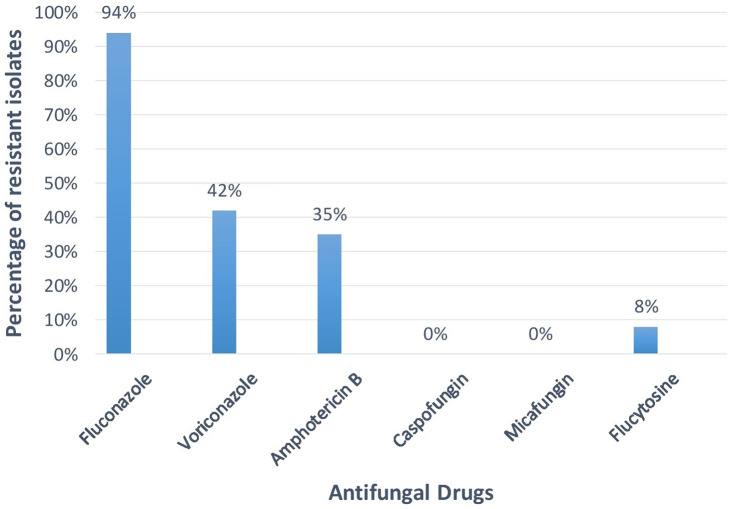
Resistance pattern of *Candida auris* with different antifungal drugs.

Figure 3 highlights the antifungal susceptibility pattern of *C. auris* isolates (n = 10).

## Discussion

This study was conducted to elucidate the complete current epidemiology of candidemia cases in a tertiary-care teaching hospital in Jaipur. Among all blood cultures received from suspected sepsis cases, *Candida* spp were isolated from 95 (2.8%), with a steady increase in the number of cases from July 2017 to June 2020. Similar prevalence rates have been documented in several studies conducted in different parts of country.^12–14^ The predominance of candidemia in male patients, as observed in the current study, was also observed by Bhattacharjee et al in 2016.^15^

The prevalence of candidemia was more common in ICUs than in wards, and the difference was statistically significant (*P* < .001). Patients admitted to an ICU occupy a complex ecological niche that facilitates colonization and subsequent development of invasive candidiasis. These factors include exposure to high-end antibiotic therapy, invasive procedures, presence of indwelling devices, comorbidities, etc. In our study, the most common risk factor associated with candidemia was long-term antibiotic therapy (68%), followed by the presence of a central venous catheter (49%). These results agree with those of other studies by Xess et al,^14^ Giri et al,^16^ and Chowta et al.^17^ Prolonged use of broad-spectrum antibiotics alter commensal gut flora, which plays an important role in prevention of fungal overgrowth. The propensity of NAC species to form biofilms on catheters and other prosthetic devices exposes these patients to a further high risk of candidemia.

Isolation of NAC from candidemia cases has been steadily increasing over the past few years. NACs are known to be responsible for higher mortality rates due to increased virulence and reduced susceptibility to antifungal drugs. Our results reflect current trends of *C. tropicalis* (38%) as the most predominant species, followed by *C*. *parapsilosis* (18%). The ARTEMIS antifungal surveillance program also noted a decreasing trend in the isolation of *C. albicans* and an increase in NACs such as *C. tropicalis* (from 4.6% in 1997 to 7.5% in 2003) and *C. parapsilosis* (from 4.2% in 1997 to 7.3% in 2003).^18^ Similar observations have been reported by studies across India.^3,5,16,19–21^ The most probable reason for this shift seems to be excessive use of fluconazole, which has led to a survival advantage for resistant species like NAC.

A noteworthy finding in our study is the isolation of *C. auris* from 10 cases (11%), ranking it the fourth most predominant isolate among all candidemia cases. This finding agrees with another recent study from a tertiary-care hospital in New Delhi where *C. auris* is the most common isolate, responsible for 39.9% cases.^22^ In the study by Rudramurthy et al^9^ involving 27 ICUs across India, 74 isolates of *C. auris* were reported, making it the fifth most common cause of candidemia.^9^ The emergence of this fungus is very concerning because it spreads in the hospital rapidly, it is resistant to multiple classes of antifungals, and it causes severe infections in critically ill patients. Another major issue is that the commonly employed phenotypic commercial system cannot identify this fungus accurately. Accurate identification of this yeast is of paramount importance when formulating a therapeutic plan. We hypothesize that this misidentification by conventional techniques could be the reason why only few studies from India have reported *C. auris.* Nevertheless, there is a need for clinicians to be aware of and prepared for any potential outbreak by this multidrug-resistant fungus. Notably, infection control practices are firmly in place and this situation is closely monitored in our hospital.

With wider and inappropriate use of antifungal drugs, antifungal resistance is likely to continue to increase. This trend will increase treatment costs in resource-poor countries like ours, where healthcare infrastructure is already strained. In our study, although NAC isolates were more resistant to antifungals than was *C. albicans*, the difference was statistically significant only for amphotericin B (*P* = .03). Similar findings have been reported in another study from northeastern India by Sabhapandit D et al.^23^

Resistance to fluconazole has been observed in 38% of isolates, with NACs being more resistant than *C. albicans* (36% vs 2%). This finding is of concern because alternative drugs like echinocandins or amphotericin B are more expensive. Kothari et al^24^ from New Delhi have also reported 36% fluconazole resistance in candidemia isolates. Similar levels of resistance have been reported by other researchers as well.^16,20,25,26^ Resistance to amphotericin B has been observed in 10.5% isolates. Similar rates of resistance have been reported in other contemporary studies as well.^23,26^ Resistance to amphotericin B was significantly high in NAC isolates in our study.

A ray of hope is that no echinocandin resistance has been observed in our study isolates. Among all antifungals, echinocandins are considered superior because of their broad spectrum of action and fewer side effects. Their high cost and limited availability, however, do not make them an easy choice for resource-constrained countries. Few studies have reported resistance to echinocandins^19^; hence, they may be considered the drug of choice for azole-resistant isolates.

Among the 95 *Candida* isolates in our study, resistance to 5-FC was observed in only 4.21%. A wide variation in resistance pattern for 5-FC from various studies in India has been reported, ranging from 11.46% to 37%.^12,27^ This pattern is probably related to difference in usage of this antifungal agent in different settings. Resistance to 5-FC is less likely to develop if it is used in combination with other antifungal drugs.

Amongst the *C. auris* strains (n = 10), resistance to fluconazole, voriconazole, amphotericin B and 5-FC was observedin 94%, 35%, 35% and 8% isolates respectively (Fig. 3). No resistance was noted for echinocandins. A multicenter study by Chowdhary et al^28^ has highlighted ∼90% azole resistance in *C. auris* isolates.^28^ A recent study by Shastri et al^22^ also reports ∼97% fluconazole resistance in *C. auris* isolates.^22^

The study had several limitations. First, the retrospective design of study may have prevented us from identifying all risk factors related to candidemia cases. With the onset of the current coronavirus 2019 (COVID-19) pandemic, the institute was converted into a designated COVID-19 care center. Maximum resources were diverted towards catering to the needs of COVID-19 patients. Therefore, this data may be a slight under representation especially in later one-thirdpart of study period. Nevertheless, our results provide clear insight into the prevalence, epidemiology, and antifungal susceptibility pattern of candidemia in our setting during contemporary times.

Candidemia continues to be a considerable cause of morbidity and mortality. The shift in epidemiology and increasing resistance to antifungals emphasize the need for constant monitoring of these cases. The clinical importance of species-level identification of etiological agents of candidemia cannot be overemphasized. *C. auris* is an emerging fungal pathogen, and the medical community needs to be on high alert regarding this yeast. The insights obtained from this study will be helpful in guiding clinical management and developing antifungal stewardship guidelines.
